# Perspectives for Developing New Tuberculosis Vaccines Derived from the Pathogenesis of Tuberculosis: I. Basic Principles, II. Preclinical Testing, and III. Clinical Testing

**DOI:** 10.3390/vaccines1010058

**Published:** 2013-01-25

**Authors:** Arthur M. Dannenberg, Bappaditya Dey

**Affiliations:** 1 Department of Environmental Health Sciences, Johns Hopkins Bloomberg School of Public Health, Baltimore, MD 21205, USA; 2 Department of Molecular Microbiology and Immunology, Johns Hopkins Bloomberg School of Public Health, Baltimore, MD 21205, USA; 3 Department of Epidemiology, Johns Hopkins Bloomberg School of Public Health, Baltimore, MD 21205, USA; 4 Department of Pathology, Johns Hopkins School of Medicine, Baltimore, MD 21231, USA; 5 Center for Tuberculosis Research, Johns Hopkins School of Medicine, Baltimore, MD 21231, USA; 6 Howard Hughes Medical Institute, Chevy Chase, MD 20815, USA; E-Mail: bdey1@jhmi.edu

**Keywords:** tuberculosis, TB perspectives, TB pathogenesis, TB immunology, TB vaccines, delayed-type hypersensitivity, cell-mediated immunity, TB in rabbits, TB in mice, TB in guinea pigs, TB in humans, TB vaccine clinical trials, TB latency, TB reactivation

## Abstract

**Part I. Basic Principles.** TB vaccines cannot prevent establishment of the infection. They can only prevent an early pulmonary tubercle from developing into clinical disease. A more effective new vaccine should optimize both cell-mediated immunity (CMI) and delayed-type hypersensitivity (DTH) better than any existing vaccine. The rabbit is the only laboratory animal in which all aspects of the human disease can be reproduced: namely, the prevention of most primary tubercles, the arrestment of most primary tubercles, the formation of the tubercle’s solid caseous center, the liquefaction of this center, the formation of cavities and the bronchial spread of the disease. In liquefied caseum, virulent tubercle bacilli can multiply extracellularly, especially in the liquefied caseum next to the inner wall of a cavity where oxygen is plentiful. The bacilli in liquefied caseum *cannot* be reached by the increased number of activated macrophages produced by TB vaccines. Therefore, new TB vaccines will have little or no effect on the extracellular bacillary growth within liquefied caseum. TB vaccines can only increase the host’s ability to stop the development of new TB lesions that arise from the bronchial spread of tubercle bacilli from the cavity to other parts of the lung. Therefore, effective TB vaccines do not prevent the reactivation of latent TB. Such vaccines only control (or reduce) the number of metastatic lesions that result *after* the primary TB lesion was reactivated by the liquefaction process. (Note: the large number of tubercle bacilli growing extracellularly in liquefied caseum gives rise to mutations that enable antimicrobial resistance—which is a major reason why TB still exists today). **Part II. Preclinical Testing.** The counting of grossly visible tubercles in the lungs of *rabbits* after the inhalation of virulent human-type tubercle bacilli is the most pertinent preclinical method to assess the efficacy of new TB vaccines (because an effective vaccine will stop the growth of developing tubercles before while they are still microscopic in size). Unfortunately, rabbits are rarely used in preclinical vaccine trials, despite their relative ease of handling and human-like response to this infection. Mice do not generate an effective DTH response, and guinea pigs do not generate an effective CMI response. Only the rabbits and most humans can establish the proper amount of DTH and CMI that is necessary to contain this infection. Therefore, rabbits should be included in all pre-clinical testing of new TB vaccines. New drugs (and/or immunological procedures) to reduce liquefaction and cavity formation are urgently needed. A simple intradermal way to select such drugs or procedures is described herein. **Part III. Clinical Testing.** Vaccine trials would be much more precise if the variations in human populations (listed herein) were taken into consideration. BCG and successful new TB vaccines should *always* increase host resistance to TB in naive subjects. This is a basic immunological principle. The efficacies of new and old TB vaccines are often not recognized, because these variations were not identified in the populations evaluated.

## 1. Introduction

These *Perspectives* address both preclinical TB in animal hosts and clinical TB in human populations. It is a guide to how new TB vaccines can be more effectively evaluated in each group. It is *not* a review of new TB vaccines or the mechanisms in which they may reduce the prevalence or morbidity of TB in the world.

## 2. Basic Principles of Resistance and Lack of Resistance to *in Vivo M. tuberculosis* Growth

### 2.1. How TB Vaccines Work

A TB vaccine cannot prevent the establishment of a primary pulmonary tuberculous lesion. The establishment of a primary lesion depends on the activation state of the alveolar macrophage (AM) that ingested the inhaled bacillus, *i.e*., whether the AM was activated nonspecifically (by dust and benign microorganisms) to inhibit the intracellular growth of an inhaled tubercle bacillus. A sufficiently activated AM normally clears the infection, whereas a poorly activated AM allows the bacillus to multiply intracellularly, establishing a primary lesion. The pulmonary AM population contains highly activated AM and poorly activated AM [1]—mostly depending on how long these AM have resided in the alveoli. Many poorly activated AM had arrived there recently.

The bacilli (multiplying in poorly activated AM) and these AM themselves release chemotactic factors that cause the migration and accumulation of blood-derived monocyte/macrophages and lymphocytes—establishing a primary tubercle. The initial AM (containing multiplying tubercle bacilli) eventually bursts. The bacilli are then ingested by the accumulating non-activated macrophages from the blood stream [2] in which the bacilli continue to multiply. Lurie called this *the stage of symbiosis*, because both the number of macrophages and number of tubercle bacilli increase at the site [3]. Bacillary multiplication at this stage of tubercle development is stopped by delayed-type hypersensitivity (DTH) to the tuberculin-like products of the bacillus [4]. The DTH response acts in two ways: (a) by directly killing the infected macrophages by CD8^+^ T-cells and thrombosis of the macrophages’ blood supply [5] and (b) by enhancing the local accumulation of lymphocytes and macrophages [4,6,7]. Simultaneously, CMI develops and activates the local macrophages by antigen-specific Th1 lymphocytes [8], so that they can prevent the intracellular growth of ingested bacilli and even kill these bacilli. At this stage, the combination of DTH and CMI can arrest the lesion.

In individuals vaccinated with effective TB vaccines, lesion progression is stopped earlier, because DTH and CMI are induced more rapidly. This rapid recall would prevent developing TB lesions from reaching X-ray visible size. In other words, effective TB vaccines can prevent clinically apparent disease.

Both DTH and CMI are caused by an antigen-specific Th1 lymphocyte population that activates macrophages within tuberculous lesions [1,6,7,9,10,11]. These locally activated macrophages ingest and kill the live tubercle bacilli that they encounter. If many activated macrophages are present, a developing TB lesion is arrested.

In brief, good TB vaccines work by more rapidly stopping the progression of small developing pulmonary tubercles. These vaccines do so (a) by DTH, *i.e*., the more rapid killing of macrophages that contain too many tubercle bacilli for CMI to inhibit—thereby causing solid caseous necrosis in the center of the tubercle in which the growth of tubercle bacilli is inhibited and (b) by CMI, *i.e*., the more rapid activation of the macrophages that surround the caseous center—so that any ingested bacillus that escapes from the solid caseous center does not grow. Therefore, in the vaccinated host, early developing TB lesions will be arrested sooner (than in the non-immunized host) and never reach clinically overt disease.

### 2.2. Differences between Delayed-Type Hypersensitivity (DTH) and Cell-Mediated Immunity (CMI)

Both DTH and CMI are cell-mediated immune responses mediated by Th1 antigen-specific lymphocytes [8]. The main difference between them is the concentration of antigen required to elicit the host response [9]. After all, delayed-type hypersensitivity (DTH) is what the name signifies, *i.e*., an increased sensitivity to the low concentrations of the antigens that elicit it.

The classic DTH reaction is the tuberculin reaction, which is an inflammatory reaction—usually measured two days after an intradermal injection of tuberculin, *i.e*., 5 tuberculin units (0.0001 mg of PPD). At two days, this dose causes a minimal inflammatory reaction (with induration, if any, of less than 10 mm in immunocompetent individuals who never had a pulmonary tubercle). This dose causes a larger induration in individuals who have an arrested a pulmonary tubercle. And, this dose causes a still larger induration (often with a necrotic center) in highly tuberculin-sensitive individuals (probably in individuals whose pulmonary lesion(s) are still more active).

CMI seems to be elicited with much higher antigen concentrations than those present in tuberculin preparations. CMI activates macrophages in tuberculous lesions. These activated macrophages kill (or stop the growth of) the tubercle bacilli that they ingest. Yet, very low concentrations of tuberculin can also activate macrophages (*i.e*., produce CMI) [12], and high concentrations of CMI antigens can probably cause tissue necrosis.

In brief, the low concentration at which DTH antigens elicit a reaction seems to be the main difference between DTH and CMI. DTH kills macrophages containing more tubercle bacilli than CMI can inhibit. In doing so, DTH forms the solid caseous center of the tubercle—within which the bacillus cannot multiply. DTH antigens and CMI antigens cause similar tissue reactions. It is only the effective concentration of each antigen that identifies the category to which that antigen belongs. As discussed in Part II below, DTH and CMI must be developed in the proper amount and proper proportion to effectively control the growth of tubercle bacilli in hosts infected with *M. tuberculosis *(*M. tbc*). Vaccines that do this will provide the best protection against clinically active disease. 

### 2.3. Formation of Pulmonary Cavities within Which Tubercle Bacilli Grow and Evade the Immune System

In about 95% of the human population, the primary pulmonary tubercle is arrested—often as a minute lesion with a solid caseous center with or without calcification. These individuals have what is called *latent TB*, because with decrease host resistance, such as in those infected with HIV, the disease becomes active. In tuberculin-positive individuals, latent TB lesions are kept arrested by an active host immune response, *i.e*., by the macrophages (that surround the solid caseum) becoming activated wherever tubercle bacilli (or their antigens) stimulate local Th1 lymphocytes. 

The remaining 5% of the human population falls into two categories: (a) the childhood-type of TB (such as miliary TB) in which the disease disseminates by the hematogenous (and lymphogenous) routes and (b) adult-type TB in which liquefaction of the solid caseous center occurs that is frequently followed by cavity formation and the bronchial spread of the disease [3,6,13,14]. In the adult type, the formerly dormant tubercle bacilli multiply in the liquid menstruum (for the first time extracellularly), sometimes reaching a very large number (in which mutations causing antimicrobial resistance may occur). The tuberculin-like products produced by such a large number of tubercle bacilli can destroy the protective surrounding TB granulation tissue (containing activated macrophages) and can erode the wall of a nearby bronchus, forming a pulmonary cavity when the liquefied caseum is discharged into the airway.

### 2.4. TB Vaccines Cannot Prevent Reactivation of Latent TB

Increasing host immunity by a TB vaccine increases the number macrophages and their speed of activation, but these host defense cells cannot survive in liquefied (or solid) caseum (probably because of the toxic and allergenic bacillary products present). Therefore, in hosts that have an arrested primary pulmonary tubercle, TB vaccines cannot stop the major clinical cause of reactivation of the adult disease, *i.e*., the liquefaction of solid caseum. In fact, such liquefaction may even be enhanced by the increased numbers of activated macrophages (and their hydrolytic enzymes) as a result of vaccination (discussed in Item 1.6, below).

In adult-type TB, vaccines can only increase the host’s ability to stop the development of new tuberculous lesions that arise from the bronchial spread of tubercle bacilli from the cavity to other parts of the lung. Therefore, effective TB vaccines do not prevent the reactivation of latent TB. Such vaccines only control (or reduce) the number of metastatic lesions that result *after* the primary TB lesion was reactivated by the liquefaction process.

In other words, TB vaccines can reduce (or control) clinically reactivated tuberculosis by arresting the development of secondary pulmonary lesions arising from bacilli spread via the airways from the primary cavity lesion. Vaccines cannot prevent the activation of latent TB (which is due to the liquefaction of the solid caseous center of the primary lesion). In fact, TB vaccines may even enhance the liquefaction process.

### 2.5. Mycobacterial Drug Resistance, Liquefaction of Solid Caseum and Cavity Formation

Chemotherapeutic drug regimens are sometimes ineffective, because of the development of drug resistance in the mycobacterial population. Such drug resistance almost always occurs in the tubercle bacilli that are growing extracellularly in liquefied caseum [15]. The most rapid growth occurs in the inner wall of a pulmonary cavity, where the highest amount of oxygen is present [15], and minimal (if any) growth occurs in solid caseum. Since liquefaction and cavities do not usually occur in tuberculous mice, this species would be less pertinent for evaluating TB drug regimens for their ability to prevent the development of drug-resistant mycobacteria. Rabbits develop cavities more readily than even guinea pigs, and therefore, rabbits would be the best species to use for this purpose [15].

To date, the effect TB vaccines have on the development of liquefaction and cavity formation has not been investigated. Such knowledge would be most applicable to vaccines used to boost immunity in individuals who have an arrested primary TB lesion. Drugs (or immunological procedures) to inhibit liquefaction of solid caseum (and therefore cavity formation) would greatly aid the present day antimicrobial treatment of tuberculosis. Such drugs could be evaluated in the skin model of liquefaction presented in reference [15] (see Part 2, Item 2.5, below).

### 2.6. A Warning on Using TB Vaccines in Humans Who Have Already Arrested TB Lesions

TB vaccines (especially BCG) are usually administered to only tuberculin-negative individuals. However, a proportion of these tuberculin-negative individuals would become tuberculin-positive if the skin test was repeated after a few weeks. This response is called the tuberculin “booster reaction” or tuberculin “recall phenomenon” [16,17]. In other words, these tuberculin-negative individuals were really tuberculin-positive with a low expanded antigen-specific T-cell population that was increased by boosting.

The identification of such individuals is usually omitted in clinical trials and is one of the reasons why vaccine trials fail to show differences between control and vaccinated groups. (Individuals who produce a booster reaction are already immunized by a natural TB infection that they arrested).

A danger exists in vaccinating such individuals. Specifically, many of these individuals have a small arrested tubercle with a solid caseous center. Liquefaction of such caseous centers (and cavity formation) occurs more readily in tuberculin-positive rabbits than in tuberculin-negative ones [15], apparently because more activated macrophages (with high levels of hydrolytic enzymes) surround the solid caseum. Therefore, vaccinating tuberculin-positive individuals might hasten the liquefaction of an arrested (dormant) TB lesion (with subsequent cavity formation). In other words, vaccinating individuals with an arrested TB lesion might sometimes activate this disease. In fact, repeated injections of BCG in tuberculous mice actually caused necrosis in the pulmonary lesions and increased the severity of the disease [18]. Mice are a species in which such necrosis is rare.

Note: Tuberculin skin tests do not seem to contain sufficient antigen to stimulate the liquefaction process, but no studies have been made on this possibility. Repeated tuberculin skin-testing has never been shown to produce tuberculin positivity in naive recipients. Yet, BCG vaccination routinely does so. These facts suggest that tuberculin itself is a minimal antigenic stimulus compared to those produced by the intact tubercle bacilli (and some antigenic fractions thereof) that are present in effective TB vaccines.

## 3. Preclinical Testing of New TB Vaccines in Mice, Guinea Pigs and Rabbits

### 3.1. Tuberculosis in Humans, Mice, Guinea Pigs, Rabbits and Non-Human Primates

In humans and rabbits, most early pulmonary TB lesions produced by inhaled virulent human-type tubercle bacilli are arrested by the developing immune response [9]. However, in mice and guinea pigs, most of these pulmonary lesions continue to enlarge until the host dies [9]. In other words, humans and rabbits usually develop an *effective* immune response and mice and guinea pigs usually do not. The immunity of nonhuman primates (to virulent human-type tubercle bacilli) seems to be well below that of rabbits and modern human beings [9]. Probably, the most genetically susceptible humans have long since been killed off by this disease.

Rabbits and human beings control the growth of inhaled virulent human-type tubercle bacilli better than any other species. For rabbits, about 300 to 3,000 of these bacilli must be inhaled to establish a single visible primary tubercle [9]. For humans, about 20 to 200 of these bacilli must be inhaled to establish a single visible primary tubercle (Richard L. Riley, personal communication described in reference 9 on pages 224–226). Such tubercles are established in mice, guinea pigs and nonhuman primates with an average inhaled dose of only 10 to 30 of these bacilli [9]. This number for cynomolgus monkeys was not available [9].

A good immune response amount requires the host to develop the correct amount of *both* delayed-type hypersensitivity (DTH) (*i.e*., tuberculin-like sensitivity) and cell-mediated immunity (CMI) (*i.e*., local macrophage activation), so that DTH and CMI can work together to inhibit the growth of tubercle bacilli [4,9]. Tuberculous mice develop only weak DTH, but develop good CMI [9]. Tuberculous guinea pigs develop good DTH, but develop only weak CMI [9]. In other words, mice are a poor species to recognize DTH-producing antigens, and guinea pigs are a poor species to recognize CMI-producing antigens. Therefore, each of these laboratory animals will not accurately reflect the DTH and CMI responses to TB vaccines that is found in humans and rabbits (which are species that develop both good DTH and good CMI responses).

Several TB antigens promote DTH responses, whilst others induce CMI. Moreover, different animal species respond to the different antigens of the tubercle bacillus in different ways—which is clearly demonstrated when TB in mice, guinea pigs, rabbits and humans are compared [9]. Using both mice and guinea pigs to evaluate new TB vaccines would be much better than using either species alone. However, since the DTH and CMI responses of rabbits more closely resemble those found in human beings, the rabbit is the most relevant laboratory animal for evaluating DTH and CMI responses simultaneously. Mice, guinea pigs, rabbits and humans will each respond to each antigen in TB vaccines in their own manner. Since no animal responds exactly as humans do, new TB vaccines should be evaluated in all three laboratory animal species (and perhaps even in nonhuman primates) before expensive clinical trials are undertaken.

### 3.2. Balancing DTH and CMI

The correct balance (or ratio) of DTH and CMI is required for a new vaccine to be better than BCG. Table 1 lists the DTH (*i.e*., the PPD skin test) and CMI (*i.e*., the interferon-gamma production by peripheral blood lymphocytes) produced in humans by many candidate TB vaccines now in clinical trials [19]. All of these vaccines increased the IFN-gamma response, and almost all of them converted the PPD skin test.

**Table 1:** Protective efficacy, delayed-type hypersensitivity (DTH) response and cell-mediated immunity (CMI) response induced by current TB vaccines in preclinical and in clinical trials.

Vaccine in clinical trials	Type of vaccine	Protection in animal models compared to BCG vaccine			Human PPD response (DTH)	Human IFNγ response (CMI)
		Mice	Guinea pigs	Rabbits		
**Phase-III**						
***M. vaccae***	Inactivated whole cell non‐TB mycobacterium	Y	ND	ND	+/−	Y
***Mw [M. indicus*** ***pranii* (MIP)]**	Whole cell saprophytic non‐TB mycobacterium	Y	Y	ND	+	Y
**Phase II**						
**MVA85A/AERAS** **‐485**	Modified vaccinia Ankara vector expressing *M. tbc* Ag 85A	Y	Y	ND	+	Y
**AERAS** **‐** **402/Crucell** **Ad35**	Replication‐deficient adenovirus 35 vector expressing *M. tbc* Ags 85A, 85B, TB10.4	NA	ND	ND	+	Y
**M72 + AS01**	Recombinant protein composed of a fusion of *M. tbc* Ags Rv1196 and Rv0125 & adjuvant AS01	EQ	EQ	EQ	+	Y
**Hybrid** **‐I + IC31**	Adjuvanted recombinant protein composed of *M. tbc* Ags 85B and ESAT‐6 [18,19,20,21,22]	EQ	EQ	ND	+	Y
**VPM 1002**	rBCG Prague strain expressing listeriolysin and carries a urease deletion mutation	Y/EQ	ND	ND	+	Y
**RUTI **	Fragmented *M. tbc* cells	Y	Y	ND	ND	Y
**Phase-I**						
**rBCG30**	rBCG Tice strain expressing *M. tbc* Ag 30 kDa 85B	Y	Y	ND	+	Y
***M. smegmatis ***	Whole cell extract	NA	NA	NA	NA	Y
**AdAg85A**	Replication‐deficient adenovirus 5 vector expressing *M. tbc* Ag 85A	Y	Y	ND	+	Y
**Hybrid** **‐I+CAF01**	Adjuvanted recombinant protein (*M. tbc* Ags 85B and ESAT‐6)	EQ	ND	ND	NA	Y
**Hybrid 56 + IC31**	Adjuvanted recombinant protein *M. tb*c Ags 85B, ESAT‐6 and Rv2660)	Y	ND	ND	+	Y
**HyVac4/** **AERAS** **‐404, +IC31**	Adjuvanted recombinant protein (fusion of *M. tbc* Ags 85B and TB10.4)	EQ	EQ	ND	+	Y
**ID93/GLA-SE**	Subunit fusion protein composed of 4 *M. tbc* Ags	EQ	EQ	ND	+	Y
**AERAS** **‐422**	Recombinant BCG expressing mutated PfoA andoverexpressing *M. tbc* Ags 85A, 85B and Rv3407	Y	ND	ND	ND	Y

Phase I, II and III, early, intermediate and late tests; BCG, Bacille Calmette-Guérin; Y, Better than BCG; EQ, Equal to BCG; ND, Not determined; NA, Not Available; +, positive. In *preclinical *testing, most of these new TB vaccines protected mice and guinea pig equally or better than BCG. (However, very few of these vaccines have been evaluated in the rabbit model.) In *clinical* testing, most new TB vaccines produced both Purified Protein Derivative (PPD) skin-test sensitivity (DTH) and antigen-specific peripheral blood IFN-gamma production (CMI) in humans receiving the new vaccines. Adapted from [19].

Table 1 also lists the protection (when available) of mice, guinea pigs and rabbits produced by these new TB vaccines when they were compared to the protection produced by BCG vaccine. Several of the new vaccines gave better protection than BCG, and others were just equivalent to BCG in the species tested. However, very few of the vaccine candidates were tested in the rabbit model.

DTH and CMI are responses to many antigens. PPD (Seibert’s Purified Protein Derivative of Koch’s Old Tuberculin) contains several antigens that are active in producing the tuberculin skin test [20], and many antigens that produce CMI are being evaluated as improvements or additions to live BCG vaccines [21,22,23]. As discussed above, mice, guinea pigs and rabbits develop different degrees of DTH and CMI when infected with virulent tubercle bacilli. Individual humans also vary in their DTH and CMI responses to tubercle bacilli. Such species and individual differences determine the balance of DTH and CMI produced in the host by new vaccines, and this balance determines how the TB-infected host handles the disease. 

Insight into the balance of DTH and CMI is best provided by aerosol infection of rabbits with virulent *M. tbc*. Pulmonary TB in this animal species matches pulmonary TB in humans better than it does in any other animal species. Both rabbits and humans arrest most early developing pulmonary tubercles, and no other laboratory animal species does so.

Insight into the balance of DTH and CMI is best provided by aerosol infection of rabbits with virulent *M. tbc*. Pulmonary TB in this animal species matches pulmonary TB in humans better than it does in any other animal species. Both rabbits and humans arrest most early developing *M. tbc* pulmonary tubercles, and no other laboratory animal species does so.

Quantitation of the tuberculin skin tests (DTH) and IFNγ blood tests (CMI) could be used to characterize the immune response of persons with or without active TB.

### 3.3. Producing and Counting Primary Pulmonary Tubercles in Rabbits

Pulmonary tubercle counts in rabbits are rarely used to assess the efficacy of new TB vaccines, because most TB investigators do not have facilities to expose rabbits to aerosols containing virulent tubercle bacilli. Existing mouse aerosol exposure chambers can expose 50 mice at a time, but are too small to expose rabbits. Existing guinea pig aerosol chambers can expose 18 guinea pigs at once, but can only house six rabbits for simultaneous exposure*.*

Lurie (using the Wells apparatus) [24] exposed six rabbits at a time. The rabbits were placed loosely in cloth bags (to restrict their activity) and then in metal cylinders (arranged like spokes of a wheel), with the head of each rabbit protruding into the aerosol chamber. Dannenberg [25,26,27] had his rabbits exposed individually at Ft. Detrick (by M. Louise M. Pitt) to 12 aerosols produced from aliquots of a single culture of virulent tubercle bacilli. Each rabbit was again placed in a loose bag and hand-held with its head protruding into the aerosol chamber for exactly 10 minutes. Both Lurie and Pitt collected samples of the aerosol with an impinger. The samples were then cultured in various dilutions to determine the number of viable tubercle bacilli in each aerosol. The dose of the bacilli inhaled by each rabbit was calculated from its respiratory rate. Tubercle counting is currently used by the Gilla Kaplan [28] and the William Bishai groups [25,26,27] for pulmonary TB studies, but they are not (to our knowledge) using the method to evaluate new TB vaccines. The Kaplan group uses a nose-only exposure system [28], and the Bishai group [26,27] currently uses a Madison chamber that is usually used to infect guinea pigs.

Different laboratories count tubercles in different ways. Lurie [3] euthanized the rabbits containing five-week old pulmonary tubercles and fixed their lungs by inflating them with 10% formalin (4% formaldehyde) via the trachea. A week or so later, he dissected each tubercle from the lungs and counted them. The number of tubercles can be almost as accurately assessed by palpation of unfixed lungs (so that the unfixed lungs can be cultured for live tubercle bacilli). Surface tubercles can be counted to estimate their total number with about 80% accuracy. 

Recently, the number of pulmonary tubercles in live rabbits has been estimated by combined computer-assisted tomography (CT) and positron emission tomography (PET) (again with about 80% accuracy) [29,30,31]. This method provides an opportunity and to monitor the progress of TB lesions in the lungs of living rabbits during the whole course of this disease and also provides an estimate of lung inflammation. Since this method detects the amount of inflammation and consolidation in TB lesions in the lungs of living rabbits, it can be used to study the liquefaction and cavity formation as they develop [31]. Use of combined PET-CT imaging has huge potential to improve the evaluation of vaccine efficacy in rabbits, as well as in other animal models. 

### 3.4. M. tbc Virulence Factors

This is the age of molecular microbiology in which the genome of microorganisms is providing clues to their virulence, as well as guides for the development of new antimicrobial agents. The genomic sequence of *M. tbc* is now known [32] and this sequence enables the identification of products that *M. tbc* manufactures. The genome of *M. tbc* transcribes two product categories: (a) those that maintain the life of the bacillus and allow it to reproduce wherever it thrives and (b) those that enable it to withstand the innate and acquired (adaptive) defenses of the host. Here are a few examples of the latter category.

(a) Phthiocerol dimycocerosate (PDIM), which makes the mycobacterial cell wall resistant to destruction by macrophages [33], (b) ESAT-6, produced by the RD1 gene region that (among other functions) induces the recruitment of non-activated macrophages in which intracellular bacillary growth readily occurs [34]—the absence of ESAT-6 contributes to the avirulence of both BCG (the widely used TB vaccine) and H37Ra (a common avirulent laboratory strain, (c) cell surface lipids that enable *M. tbc* to survive within the phagosome and prevent its acidification and maturation [35] and (d) factors that enable the bacillus to survive in solid caseous necrosis in the guinea pig model of tuberculosis [36,37].

Antimicrobial drugs and immunological defenses that inhibit the survival and growth of *M. tbc* may also be effective against a variety of other microorganisms. However, antimicrobial drugs and immunological defenses that reduce the ability of *M. tbc* to survive for years in solid caseum are probably more specific for this microorganism.

Finally, human beings vary considerably in their response to different microbial antigens. A strong immunological defense against specific *M. tbc* antigens (found in most individuals) is usually able to keep the disease arrested for the person’s lifetime, whereas a weak immunological defense against such antigens (found in some individuals) would allow the *M. tbc* that escape from an arrested TB lesion to cause clinical disease.

The genomic field is still in its infancy, and many more factors involved in host-parasite interactions will be discovered in the next few years.

### 3.5. Future Research Directed towards TB Latency and Reactivation

To our knowledge, no laboratory is studying the process by which solid caseous tissue is produced in the center of developing tubercles, and no laboratory is studying the factors that cause this solid caseum to liquefy and forms cavities. Yet, these two processes are responsible, respectively, for the latency and the reactivation of tuberculosis in human populations. The rabbit is the only easily accessible laboratory animal in which both of these processes are readily produced [9]. Mice rarely form caseous tubercles, never form liquefied caseum and never form cavities [9]. Guinea pigs do form solid caseum, but only rarely form liquefied caseum and cavities [9]. (Before cavities develop, guinea pigs usually die of the childhood form of TB that is hematogenously spread rather than bronchial spread).

In the rabbit, most early developing tubercles caused by virulent *M.tbc* (*i.e*., virulent human-type tubercle bacilli), regress and never reach visible size, because (as stated above in Item 2.1) 300 to 3,000 *M. tbc* must be inhaled by rabbits to form one tubercle that is visible five weeks afterwards [9]. In rabbits, the majority of the pulmonary tubercles caused by the *human-type* bacillus would regress when the immune process begins and would be hard to find at necropsy in tissues sections of their lungs.

To more easily study the factors involved with the development of *solid and liquefied* caseum and cavity formation in rabbits, investigators should use virulent *bovine-type* tubercle bacilli, *i.e*., virulent *M. bovis* (e.g., the Ravenel strain), rather than virulent human-type tubercle bacilli [9]. Rabbits develop a visible tubercle when less than 10 virulent *M. bovis* are inhaled, so that after inhaling a relatively large dose of virulent *M. bovis*, developing and progressing pulmonary tubercles would be rather easy to find [38,39]. With virulent bovine-type tubercle bacilli, all beginning pulmonary tubercles progress forming a solid caseous center or a liquefying caseous center that is frequently followed by cavity formation. Histological studies and immunological studies, as well as knockout gene and quantitative gene studies, could then be used to elucidate the mechanisms involved in caseation, liquefaction and cavity formation. As stated above, rabbits are the only common laboratory species in which virulent tubercle bacilli frequently liquefy solid caseum and form pulmonary cavities.

Liquefied caseum and cavities are produced readily in rabbits inhaling virulent *M. bovis* [38,39], especially if the rabbits are made tuberculin-positive by vaccines or by a previous exposure to virulent tubercle—probably (as stated above) because increased numbers of activated macrophages (containing increased levels of hydrolytic enzymes) are present. Specific inactivation of such enzymes by pharmaceuticals or by immunological procedures could stop or reduce liquefaction and cavity formation—thereby decreasing the prevalence of tuberculosis in the world.

A convenient way to evaluate methods to reduce liquefaction and cavity formation is to produce a surrogate model for these occurrences in the skin of rabbits, specifically, intradermal injections of BCG (or even killed virulent tubercle bacilli) in ascending concentrations (*i.e*., low to high amounts in 0.1 mL of diluent) [15]. Rabbits receiving effective inhibitor therapy would require a higher concentration of BCG (or dead virulent tubercle bacilli) to produce liquefaction and ulceration of the skin than would rabbits not receiving such therapy. Effective therapies could then be evaluated in the *M. bovis*-produced pulmonary model of cavity formation, just described.

## 4. Ways to Improve the Clinical Testing of New TB Vaccines (Adapted from [9])

### 4.1. Identify a Human Population That Could Benefit from Receiving a TB Vaccine

In naive populations (*i.e*., tuberculin skin-test negative), about 95% of individuals are inherently resistant to active tuberculosis, because they produce an effective immune response to the tubercle bacillus without a vaccine [40,41]. Therefore, an effective TB vaccine could only benefit about 5% of a total population. If exposed, these 5% of individuals would develop active disease and could even die from it. These individuals evidently produce an insufficient immune response and would therefore be an appropriate trial population in which to evaluate TB vaccines.

An effective TB vaccine could reduce the number of clinically active tuberculosis cases from 5% to 1% by expanding the appropriate T lymphocyte populations. In other words, the TB vaccine could now protect about 80% of this group [42,43,44,45,46]. Complete protection of every individual may never be achieved.

Note that the 95%, 4% and 1% of individuals approximate those found in populations in United States and Europe. Underlying genetic and environmental factors can dramatically shift this ratio [47]. Developing countries with a high percentage of immune-deficient individuals from HIV infection would have a different proportion in each group (see Item 3.2, below). In other words, the efficacy of TB vaccines would vary from one geographic region to another.

At present, the only way to select a population that could benefit from a better TB vaccine is to run a preliminary study with a standardize BCG strain and compare the rates of healing between several populations. Populations that healed slowly would have more individuals in the 5% group that could benefit from the vaccine. However, populations that healed quickly would have more individuals in the 95% group in which benefits from the vaccine would be unrecognizable. Several such preliminary trials need to be performed in order to establish the best way to recognize the slow and rapid healers of BCG lesions in clinical populations.

### 4.2. Identify HIV-Infected Individuals

A high prevalence of infection with HIV exists in some developing countries, especially in sub-Saharan Africa. HIV infection lowers host acquired (adaptive) immunity to the tubercle bacillus [47,48]. Therefore, HIV-infected persons would respond less well to BCG vaccination and other vaccines than would persons who are not infected with HIV. If the HIV-infected group were identified and separated from the non-HIV group, the beneficial effects of BCG and newer TB vaccines would be more easily recognized.

BCG vaccination would protect some *M. tbc*-infected, HIV-infected individuals from developing clinically active disease when the HIV only partly decreased their immune response. However, BCG vaccination would have no benefit or could even be detrimental if HIV greatly lowered their immune response. In the Karonga/Malawi BCG trial, 57% of cases of clinical tuberculosis were directly attributable to HIV infection [47,48].

### 4.3. Identify Individuals with Intestinal Helminths and/or Poor Nutrition

In developing countries, tuberculosis and intestinal worm infections often occur in the same groups of people [49]. Worm infections may cause some debilitation and also lower host resistance to tuberculosis [49]. Therefore, worm-infested populations would respond less well to BCG vaccination (and to newer TB vaccines) than would non-infested populations [49,50]. Poor nutrition would probably have a similar effect [51,52]. (If identified during these trials, individuals with helminth infections and/or poor nutrition should be given treatment, which would allow their inclusion in future trials).

### 4.4. Assess the Role of Environmental Mycobacteria

Environmental mycobacteria could have increased immunity in unvaccinated control groups [47,53,54,55], so the beneficial effects of BCG would be hard to detect, as in the Malawi trial [47] and in other inconclusive trials [41,53,54,55]. A comparison of the rates of healing of dermal BCG lesions in Europe and/or USA with those in the developing country would throw light on this possibility.

### 4.5. Perform Booster Tuberculin Skin Tests

Many individuals in tuberculin-negative groups may show a booster reaction if skin-tested again with tuberculin [16,17,56]. If they did, they would already have increased immunity to *M. tbc* from a healed natural infection. Therefore, BCG or a new TB vaccine would provide little additional benefit. Groups showing such booster reactions should not be considered tuberculin-negative.

### 4.6. Identify Antigenic Differences among TB Vaccines

Some of these vaccines (including different strains of BCG) will be more effective than others depending on their antigenic composition (discussed in [9]). *M. tb*c antigens in current clinical trials are in listed in Table 1.

### 4.7. Identify Human Genetic Differences

BCG vaccination reduced clinical tuberculosis in North American Indians by 50 to 80% [42,43,46]. This indigenous population probably had not been exposed to the tubercle bacillus for as many centuries as had the populations in Europe and Asia and, therefore, might have had more individuals who can benefit from BCG vaccination. In contrast, the trial in Chingleput, South India, showed that BCG vaccination was not beneficial [57]. This may have been because a higher percentage of the population selected belonged to the “95%” group who were naturally resistant to clinical disease.

## 5. Conclusions

This paper provides perspectives on the immunization of humans, mice, guinea pigs, rabbits and monkeys that have not usually been considered in evaluating new TB vaccines for clinical trials. These perspectives are briefly as follows.

### 5.1. DTH and CMI

Both delayed-type hypersensitivity (DTH) and cell-mediated immunity (CMI) must be produced in a host to arrest the progress of tuberculosis. DTH and CMI are similar immunological processes involving Th1 lymphocytes. However, CMI and DTH inhibit the growth of *M. tbc* by different mechanisms: CMI activates macrophages so that they inhibit the growth of *M. tbc* that they ingest. DTH kills non-activated macrophages that become overloaded with *M. tbc* and produces solid caseous necrosis in which the bacillus does not grow. (Non-activated macrophages are present in every active tuberculous lesion and may ingest and become overloaded with them).

DTH and CMI are produced by different *M. tbc* antigens and new vaccines must contain these antigens in the proper amount. Mice (infected with *M. tbc*) have weak tuberculin sensitivity (DTH) and apparently good CMI, and they usually die of the disease. Guinea pigs (infected with *M. tbc*) have good tuberculin sensitivity (DTH) and apparently weak CMI, and they also usually die of the disease. However, most humans and rabbits (infected with *M. tbc*) usually stop the progress if this disease. Therefore, we concluded that mice do not respond well to DTH-producing antigens, and guinea pigs do not apparently respond well to CMI-producing antigens. Yet, humans and rabbits (species that usually arrest the disease produced by *M. tbc*) evidently respond well to both DTH- and CMI-producing antigens.

### 5.2. Selecting the Best New TB Vaccine

In human trials, BCG vaccination has *not* been consistently beneficial. Yet, in laboratory animals, BCG has consistently increased host resistance to challenge with *M. tbc*. We propose that the rate of healing of the BCG lesions (used as a control for new vaccines in clinical trials) will identify the 95% of humans who arrest infection with *M. tbc* without the need of vaccination. In the remaining 5%, the benefits of BCG vaccination should be easier to recognize and should be more consistent with those found in laboratory animals.

The arrestment of early pulmonary tubercles by the immune process before they become clinically apparent is the very purpose of TB vaccination. Early tubercles in mice and guinea pigs are not as easily arrested, but *most* early pulmonary tubercles caused by *M. tbc* in rabbits and humans *are* arrested.

Because of expense, the tubercle counting in rabbits has been not been undertaken before starting much more expensive clinical trials. However, tubercle counting in rabbits could select the most effective new TB vaccines more precisely than any other procedure, because in rabbits (as in humans) the progress of many developing tubercles are arrested by immune forces, whereas in mice and guinea pigs, few, if any, such tubercles are arrested by such forces. Therefore, tubercle counting in rabbits should be performed before clinical trials on new TB vaccines are begun.

The antigens recognized by mice and those recognized by guinea pigs *together* may (or may not) be the same as the antigens recognized by rabbits. And, the antigens recognized by rabbits may (or may not) be the same as the antigens recognized by humans. Such differences and similarities remain to be investigated. Therefore, we urge investigators to always include rabbits along with mice, guinea pigs and, perhaps, monkeys in the preclinical testing of new TB vaccines in order to make preclinical studies more complete.

### 5.3. Critical Antigens

Vaccines containing critical antigens (possibly the Antigen 85 complex, ESAT-6 or hspX) could increase the immunity of the host to a greater extent than that produced by a natural *M. tbc* infection. Immunization with such critical antigens would increase the host’s ability to neutralize the major components of *M. tbc *that determine its virulence. Such vaccines could then be used for both TB prophylaxis and TB immunotherapy. However, only some critical antigens have so far been identified.

## References

[1] Dannenberg A.M., Burstone M.S., Walter P.C., Kinsley J.W. (1963). A histochemical study of phagocytic and enzymatic functions of rabbit mononuclear and polymorphonuclear exudate cells and alveolar macrophages. I. Survey and quantitation of enzymes and states of cellular activation. J. Cell. Biol..

[2] Dannenberg A.M. (2003). Macrophage turnover, division and activation within developing, peak and “healed” tuberculous lesions produced in rabbits by BCG. Tuberculosis (Edinb.).

[3] Lurie M.B. (1964). Resistance to Tuberculosis: Experimental Studies in Native and Acquired Defensive Mechanisms.

[4] Dannenberg A.M. (1993). Immunopathogenesis of pulmonary tuberculosis. Hosp. Pract. (Off. Ed.).

[5] Courtade E.T., Tsuda T., Thomas C.R., Dannenberg A.M. (1975). Capillary density in developing and healing tuberculous lesions produced by BCG in rabbits. A quantitative study. Am. J. Pathol..

[6] Dannenberg A.M. (2006). Pathogenesis of Human Tuberculosis: Insights from the Rabbit Model.

[7] Shigenaga T., Dannenberg A.M., Lowrie D.B., Said W., Urist M.J., Abbey H., Schofield B.H., Mounts P., Sugisaki K. (2001). Immune responses in tuberculosis: Antibodies and CD4-CD8 lymphocytes with vascular adhesion molecules and cytokines (chemokines) cause a rapid antigen-specific cell infiltration at sites of bacillus Calmette-Guerin reinfection. Immunology.

[8] Abbas A.K., Lichtman A.H. (2009). Basic Immunology: Functions and Disorders of the Immune System.

[9] Dannenberg A.M. (2010). Perspectives on clinical and preclinical testing of new tuberculosis vaccines. Clin. Microbiol. Rev..

[10] Dannenberg A.M. (1998). Lurie’s tubercle-count method to test TB vaccine efficacy in rabbits. Front. Biosci..

[11] Dannenberg A.M. (1968). Cellular hypersensitivity and cellular immunity in the pathogensis of tuberculosis: Specificity, systemic and local nature, and associated macrophage enzymes. Bacteriol. Rev..

[12] Ando M. (1973). Macrophage activation in tuberculin reactions of rabbits with primary BCG infection and reinfection. J. Reticuloendothel. Soc..

[13] Rich A.R. (1951). The Pathogenesis of Tuberculosis.

[14] Canetti C. (1955). The Tubercle Bacillus in the Pulmonary Lesion of Man: Histobacteriology and Its Bearing on the Therapy of Pulmonary Tuberculosis.

[15] Dannenberg A.M. (2009). Liquefaction and cavity formation in pulmonary TB: A simple method in rabbit skin to test inhibitors. Tuberculosis (Edinb.).

[16] Menzies D. (1999). Interpretation of repeated tuberculin tests. Boosting, conversion and reversion. Am. J. Respir. Crit. Care Med..

[17] Thompson N.J., Glassroth J.L., Snider D.E., Farer L.S. (1979). The booster phenomenon in serial tuberculin testing. Am. Rev. Respir. Dis..

[18] Cruz A., Fraga A.G., Fountain J.J., Rangel-Moreno J., Torrado E., Saraiva M., Pereira D.R., Randall T.D., Pedrosa J., Cooper A.M. (2010). Pathological role of interleukin 17 in mice subjected to repeated BCG vaccination after infection with *Mycobacterium tuberculosis*. J. Exp. Med..

[19] Tuberculosis Vaccine Candidates. http://www.stoptb.org/wg/new_vaccines/assets/documents/TB%20Vaccine%20Pipeline_rAug%202012.pdf.

[20] Seibert F.B., Crumb C., Dufour E.H. (1951). Antigenic differences in two tuberculin protein fractions. J. Infect. Dis..

[21] Kaufmann S.H., Hussey G., Lambert P.H. (2010). New vaccines for tuberculosis. Lancet.

[22] Raviglione M., Marais B., Floyd K., Lonnroth K., Getahun H., Migliori G.B., Harries A.D., Nunn P., Lienhardt C., Graham S. (2012). Scaling up interventions to achieve global tuberculosis control: Progress and new developments. Lancet.

[23] Kupferschmidt K. (2011). Infectious disease. Taking a new shot at a TB vaccine. Science.

[24] Lurie M.B., Heppleston A.G., Abramson S., Swartz I.B. (1950). Evaluation of the method of quantitative airborne infection and its use in the study of the pathogenesis of tuberculosis. Am. Rev. Tuberc..

[25] Dannenberg A.M., Bishai W.R., Parrish N., Ruiz R., Johnson W., Zook B.C., Boles J.W., Pitt L.M. (2000). Efficacies of BCG and vole bacillus (Mycobacterium microti) vaccines in preventing clinically apparent pulmonary tuberculosis in rabbits: A preliminary report. Vaccine.

[26] Manabe Y.C., Dannenberg A.M., Tyagi S.K., Hatem C.L., Yoder M., Woolwine S.C., Zook B.C., Pitt M.L., Bishai W.R. (2003). Different strains of *Mycobacterium tuberculosis *cause various spectrums of disease in the rabbit model of tuberculosis. Infect. Immun..

[27] Dorman S.E., Hatem C.L., Tyagi S., Aird K., Lopez-Molina J., Pitt M.L., Zook B.C., Dannenberg A.M., Bishai W.R., Manabe Y.C. (2004). Susceptibility to tuberculosis: Clues from studies with inbred and outbred New Zealand White rabbits. Infect. Immun..

[28] Subbian S., Tsenova L., O'Brien P., Yang G., Koo M.S., Peixoto B., Fallows D., Zeldis J.B., Muller G., Kaplan G. (2011). Phosphodiesterase-4 inhibition combined with isoniazid treatment of rabbits with pulmonary tuberculosis reduces macrophage activation and lung pathology. Am. J. Pathol..

[29] Via L.E., Schimel D., Weiner D.M., Dartois V., Dayao E., Cai Y., Yoon Y.S., Dreher M.R., Kastenmayer R.J., Laymon C.M. (2012). Infection dynamics and response to chemotherapy in a rabbit model of tuberculosis using [(1)(8)F]2-fluoro-deoxy-D-glucose positron emission tomography and computed tomography. Antimicrob. Agents Chemother..

[30] Dey B., Luna B., Miller-Jaster K., Foster B., Bagci U., Klunk M., Mollura D.J., Jain S.K., Bishai W.R. (2012). Qualitative and Quantitative Analysis of Inflammation in Pulmonary Tuberculosis in Rabbit using F18-FDG-PET/CT Imaging: A multi-Parametric Approach. Molecular Imaging of Infectious Diseases: Current Status and Future Challenges.

[31] Luna B., Kubler A., Larsson C., Klunk M., Jain S.K., Bishai W.R. (2012). Cavity Development in the Rabbit Model of Tuberculosis is Independent of Inflammation. Molecular Imaging of Infectious Diseases: Current Status and Future Challenges.

[32] Brodin P., Demangel C., Cole S.T., Jacobs W.R. (2005). Introduction to functional genomics of the *Mycobacterum*
*tuberculosis* complex. Tuberculosis and the Tubercle Bacillus.

[33] Murry J.P., Pandey A.K., Sassetti C.M., Rubin E.J. (2009). Phthiocerol dimycocerosate transport is required for resisting interferon-gamma-independent immunity. J. Infect. Dis..

[34] Volkman H.E., Pozos T.C., Zheng J., Davis J.M., Rawls J.F., Ramakrishnan L. (2010). Tuberculous granuloma induction via interaction of a bacterial secreted protein with host epithelium. Science.

[35] Russell D.G., Cole S.T., Eisenach K.D., McMurray D.N., Jacobs W.R. (2005). *Mycobacterium tuberculosis*: The indigestible microbe. Tuberculosis and the Tubercle Bacillus.

[36] Converse P.J., Karakousis P.C., Klinkenberg L.G., Kesavan A.K., Ly L.H., Allen S.S., Grosset J.H., Jain S.K., Lamichhane G., Manabe Y.C. (2009). Role of the *dosR-dosS* Two-Component Regulatory System in *Mycobacterium tuberculosis* Virulence in Three Animal Models. Infect. Immun..

[37] Jain S.K., Hernandez-Abanto S.M., Cheng Q.-J., Singh P., Ly L.H., Klinkenberg L.G., Morrison N.E., Converse P.J., Nuermberger E., Grosset J. (2007). Accelerated detection of *Mycobacterium tuberculosis *genes essential for bacterial survival in guinea pigs, compared with mice. J. Infect. Dis..

[38] Converse P.J., Dannenberg A.M., Estep J.E., Sugisaki K., Abe Y., Schofield B.H., Pitt M.L. (1996). Cavitary tuberculosis produced in rabbits by aerosolized virulent tubercle bacilli. Infect. Immun..

[39] Converse P.J., Dannenberg A.M., Shigenaga T., McMurray D.N., Phalen S.W., Stanford J.L., Rook G.A., Koru-Sengul T., Abbey H., Estep J.E. (1998). Pulmonary bovine-type tuberculosis in rabbits: Bacillary virulence, inhaled dose effects, tuberculin sensitivity, and *Mycobacterium vaccae *immunotherapy. Clin. Diagn. Lab. Immunol..

[40] Smith P.G., Moss A.R., Bloom B.R. (1994). Epidemiology of tuberculosis. Tuberculosis: Pathogenesis, Protection and Control.

[41] Bloom B.R., Fine P.E.M., Bloom B.R. (1994). The BCG Experience: Implications for future vaccines for tuberculosis. Tuberculosis: Pathogenesis, Protection, and Control.

[42] Aronson J.D. (1957). The status of BCG vaccination in the United States and Canada. Bibl. Tuberc..

[43] Aronson J.D., Aronson C.F., Taylor H.C. (1958). A twenty-year appraisal of BCG vaccination in the control of tuberculosis. AMA Arch. Intern. Med..

[44] Medical Research Council (1972). BCG and vole bacillus vaccines in the prevention of tuberculosis in adolescence and early adult life. Bull. World Health Organ..

[45] Rosenthal S.R., Loewinsohne E., Graham M.L., Liveright D., Thorne G., Johnson V. (1961). BCG vaccination against tuberculosis in Chicago. A twenty-year study statistically analyzed. Pediatrics.

[46] Aronson N.E., Santosham M., Comstock G.W., Howard R.S., Moulton L.H., Rhoades E.R., Harrison L.H. (2004). Long-term efficacy of BCG vaccine in American Indians and Alaska Natives: A 60-year follow-up study. JAMA.

[47] Crampin A.C., Glynn J.R., Fine P.E. (2009). What has Karonga taught us? Tuberculosis studied over three decades. Int. J. Tuberc. Lung Dis..

[48] Barnes P.F., Bloch A.B., Davidson P.T., Snider D.E. (1991). Tuberculosis in patients with human immunodeficiency virus infection. N. Engl. J. Med..

[49] Zevallos K., Vergara K.C., Gilman R.H., Kosek M., Yori P., Banda C., Herrera B., Valencia T., Vidal C., Meza G. (2006). Human cell-mediated immunity against *Mycobacterium tuberculosis* antigens is augmented by treating intestinal helminths. Am. J. Trop. Med. Hyg..

[50] Elias D., Wolday D., Akuffo H., Petros B., Bronner U., Britton S. (2001). Effect of deworming on human T cell responses to mycobacterial antigens in helminth-exposed individuals before and after bacille Calmette-Guerin (BCG) vaccination. Clin. Exp. Immunol..

[51] Cegielski J.P., McMurray D.N. (2004). The relationship between malnutrition and tuberculosis: Evidence from studies in humans and experimental animals. Int. J. Tuberc. Lung Dis..

[52] Liu P.T., Stenger S., Tang D.H., Modlin R.L. (2007). Cutting edge: Vitamin D-mediated human antimicrobial activity against *Mycobacterium tuberculosis* is dependent on the induction of cathelicidin. J. Immunol..

[53] Colditz G.A., Brewer T.F., Berkey C.S., Wilson M.E., Burdick E., Fineberg H.V., Mosteller F. (1994). Efficacy of BCG vaccine in the prevention of tuberculosis. Meta-analysis of the published literature. JAMA.

[54] Comstock G.W. (1994). Field trials of tuberculosis vaccines: How could we have done them better?. Control Clin. Trials.

[55] Fine P.E.M., Reichmann L.R., Hershfield E.S. (2000). BCG Vaccines and Vaccination. Tuberculosis: A Comprehensive International Approach.

[56] Comstock G.W., Woolpert S.F. (1978). Tuberculin conversions: True or false?. Am. Rev. Respir. Dis..

[57] Tuberculosis Research Centre (ICMR), Chennai (1999). Fifteen year follow up of trial of BCG vaccines in south India for tuberculosis prevention. Indian J. Med. Res..

